# Sex modifies the association between the *CLOCK* variant rs1801260 and BMI in school-age children

**DOI:** 10.1371/journal.pone.0236991

**Published:** 2020-08-12

**Authors:** Ying Meng, Barbara Lohse, Leslie Cunningham-Sabo

**Affiliations:** 1 School of Nursing, University of Rochester, Rochester, New York, United States of America; 2 Wegmans School of Health and Nutrition, Rochester Institute of Technology, Rochester, New York, United States of America; 3 Department of Food Science and Human Nutrition, Colorado State University, Fort Collins, Colorado, United States of America; Cincinnati Children’s, UNITED STATES

## Abstract

Disruption of circadian rhythms and variations in the *FTO* gene may interfere with energy homeostasis and play a role in the development of obesity. The current study assessed the association of common polymorphisms in the *CLOCK* and *FTO* genes with standardized body mass index scores (BMI z-scores) and their potential modification of the impact of a culinary nutrition and physical activity intervention in school-age children. Anthropometric measurements were collected in 121 children at the baseline and one-year follow-up of a controlled trial of a school-based culinary nutrition and physical activity intervention. Genotypes of the *CLOCK* polymorphism (rs1801260) and the *FTO* polymorphism (rs9939609) were obtained from buccal swabs. Linear mixed-effects regression was applied to evaluate the genetic association and adjust for clusters within families and schools. In our participants, obesity affected 6.6% (8/121) of the children at the baseline and 6.4% (7/109) of the children at the follow-up. The associations between the age- and sex-adjusted BMI z-scores and the two polymorphisms did not reach statistically significance. Yet, sex potentially modified the association between rs1801260 and BMI z-scores. In girls, the G allele carriers had a higher BMI z-scores at the baseline and the follow-up. These polymorphisms did not modify the effect of our culinary nutrition and physical activity intervention on BMI z-scores. Sex is a potential modifier for the association between the *CLOCK* polymorphism, rs1801260, and BMI z-scores in school-age children. Further investigation is warranted to delineate the sex-dependent role of the *CLOCK* polymorphisms in the development of childhood obesity.

## Introduction

The circadian system plays a role in the regulation of metabolic homeostasis. Human studies and animal models have implicated that disruption of circadian rhythms affects the sleep-wake cycle, eating behaviors, insulin sensitivity, and lipid metabolism [1, 2]. Therefore, circadian desynchrony may be involved in the development of obesity and metabolic disorders.

A core component of the circadian system is the clock circadian regulator (*CLOCK*) gene. This gene encodes a transcription factor that forms a heterodimer with *BMAL1* and activates the expression of subsequent circadian-network genes [1]. *CLOCK*-mutant mice have displayed hyperphagia, altered feeding pattern, increased adiposity, hyperlipidemia, impaired gluconeogenesis, and increased susceptibility of becoming obese [3–5]. In humans, polymorphisms of this gene have been associated with body mass index (BMI) and obesity in adults and adolescents [6–8]. Studies also found that sleep duration and dietary fat intake might modify these associations [9, 10]. The most studied single nucleotide polymorphism (SNP) in the *CLOCK* gene is rs1801260 [11]. Yet, the associations of this SNP with BMI, obesity have been inconsistent in adults [10, 12–20]. The minor allele of this SNP was associated with higher BMI in some studies or subgroups, such as overweight/obese adults [10, 14, 15], but lower BMI/obesity risk or no associations in other studies [12, 13, 19, 20]. Additionally, this SNP potentially modified the effect of weight loss interventions, as the minor allele carriers showed more resistance to interventions [16–18].

Childhood obesity has put children at greater risk for the development of heart disease and diabetes when they grow into adulthood [21, 22]. Identification of genetic markers related to childhood obesity and weight loss interaction would benefit precision management of high risk pediatric population. Since the *CLOCK* SNP, rs1801260, might be associated with BMI and weight loss intervention in adults, it potentially had similar effect in children. Giovaninni et al. [23] and Valladares et al. [24] assessed this SNP in school-age children, but the difference in BMI between minor allele carriers and non-carriers did not reach statistical significance.

Compared to the limited studies on the *CLOCK* SNP, the SNP, rs9939609, within the first intron of the fat mass and obesity-associated (*FTO)* gene has been widely investigated for its association with childhood obesity [25, 26]. The *FTO* gene functions as a nucleic acid demethylase and regulates DNA and RNA modifications [27, 28]. Studies have suggested that the *FTO* gene might be involved in the regulation of food intake and energy balance [29, 30]. Additionally, studies have found that the *FTO* polymorphisms were associated with the expression level of the *IRX3* gene, which could potentially link the *FTO* polymorphisms with obesity by regulating metabolic rate in the adipose tissue [30, 31]. Although the association between this *FTO* SNP and childhood obesity have been replicated, the role of this SNP in weight loss interventions was unclear [32–34].

To date, the evidence of the relationship between the *CLOCK* SNP and childhood obesity was limited, and the role of the *CLOCK* and *FTO* SNPs on weight loss interventions was unknown. Thus, the primary objective of this study was to investigate the association of the common *CLOCK* and *FTO* SNPs, rs1801260 and rs9939609, with BMI in school-age children from two school districts in one Northern Colorado county who participated in a culinary nutrition and physical activity intervention. Additionally, we assessed the potential modification effect of these SNPs on the effect of this intervention on BMI changes.

## Methods

### Recruitment

Participants had been enrolled in the Fuel for Fun study as 4th graders and were participating in a longitudinal follow-up assessment. Fuel for Fun was a multi-component, school- and family-based, cluster-randomized intervention trial (NCT02491294) designed to improve culinary skills, dietary intake, and physical activity of 4th grade youth [35]. Components included classroom cooking and tasting lessons, cafeteria promotion of foods prepared in the classroom (e.g., fruits and vegetables), inclusive games during recess, and family activities such as fun nights at school, and cooking and activity ‘fun-work’ for students to share with their family. Eight schools in two school districts in Northern Colorado participated in the study. Parents who had participated in Fuel for Fun (n = 428) were informed about the longitudinal follow-up by email, school communications, and, in a few instances, by telephone. The communication included a link to the consent forms, a buccal swab procedure video, and a survey for parents to complete, which was required for arranging youth DNA sample collection. Parents provided consent for their child’s participation and children assented before providing buccal tissue samples, height and weight, and survey data. All youth in the Fuel for Fun study whose parents had consented to DNA collection for their child and completed the survey (n = 132), assented to buccal swabs, height and weight measures and survey completion. Of these, 126 provided a usable DNA sample. Youth and their parent were provided a modest financial incentive. The Institutional Research Boards from Colorado State University and Rochester Institute of Technology approved this study.

### DNA sample and genotyping

DNA was collected by buccal swabbing that followed manufacture protocols (MME Medical Wire & Equipment, Wiltshire England). Buccal swab samples were collected at one of three sites near participating schools. Participants who could not attend any data collection event but agreed to provide a buccal sample, received DNA collection materials in the mail (n = 7) and a link to an instructional video developed by the research staff. Samples were return mailed to the University office (n = 5) for collation with onsite-collected samples. DNA extraction, purification and genotyping were performed by the Pennsylvania State University Genomics Core Facility (University Park, PA). DNA samples were isolated according to the method described by Freeman et al. [36]. SNP genotyping was performed using Thermo Fisher TaqMan^®^ Genotyping Assays (Assay ID C___8746719_20 for rs1801260 _A_G and Assay ID C_30090620_10 for rs9939609_T_A) according to the manufacture “TaqMan_SNP_Genotyping Assays” protocol (Thermo Fisher, Carlsbad, CA). TaqMan assays were performed using a QuantStudio^®^ 12K Flex Real-Time PCR System (Thermo Fisher, Carlsbad, CA). The QuantStudio^®^ 12K Flex software was used for quality control and for SNP calls. Additionally, 10% of samples were randomly chosen to be genotyped in duplicate for the assay to evaluate the accuracy of genotype calls. All duplicate genotype calls matched the genotype call of the first replicate. We used HaploReg (v4.1) and GTEx (https://www.gtexportal.org/home/) to analyze the effect of these SNPs on related genes [37].

### Anthropometric measures and covariates

Weight and height were measured by trained personnel at baseline and 12 months after baseline. Height was measured with arms hanging to the sides and looking straight ahead to the nearest tenth of a centimeter using a portable stadiometer (Model 213, SECA). Weight was measured to the nearest tenth of a kilogram using a standard scale (Model 394KLX, Health o meter) with shoes and heavy clothing (e.g., sweatshirts, jackets) removed. Age- and sex-specific standardized BMI scores (BMI z-scores) were calculated based on the growth chart developed by the US Centers for Disease Control and Prevention. Potential confounders controlled in the analyses included age (months), sex, race (white, non-white), and screen time (hours per day spent watching TV, playing videogames or using internet) collected using the validated Godin Shepherd Leisure Time Questionnaire, as previously described [35].

### Statistical analysis

Two hypotheses were assessed in this study. The first hypothesis was that the G allele of rs1801260 and the A allele of rs9939609 was associated with higher BMI z-scores, respectively. The second hypothesis was that the rs1801260 G allele and the rs9939609 A allele carriers had lower BMI z-score reductions post intervention, respectively. Therefore, the standardized BMI z-scores at baseline and 12-month follow up were the primary outcomes. The *CLOCK* and *FTO* SNPs, the culinary nutrition and physical activity intervention, and their interaction were independent variables of interest. Subjects who had missing BMI data at the baseline (n = 5) were excluded. Twelve subjects had missing BMI data at the follow-up. Their BMI z-scores at the baseline were not significantly different between the intervention group and the control group (*p* = 0.81). Therefore, these subjects were also excluded when the intervention effect was assessed for BMI z-scores at the follow-up. Covariates, including age, sex, race, and screen time were also adjusted in the statistical models. The distribution of screen time was skewed, so the variable was log-transformed. The differences of these variables between the children assigned for the treatment group and the control group at the baseline and the follow-up were assessed using t test and chi-squared test. Because participants were clustered within 113 families and eight schools, mixed-effects linear regression with random effects was applied to account for the correlations within clusters. Mixed-effects regression was used to assess the genetic association, intervention effect, and potential genetic modification effect. If the modification effect was significant, subsequent stratified analyses with mixed-effects model were conducted to identify the subgroup in which the SNP had significant association. To account for multiple testing with two SNPs, BMI outcomes at baseline and follow-up, and the interaction with the intervention, we assumed the significant p value to be less than 0.008 based on the Bonferroni correction. All analyses were conducted using STATA 13.0 (StataCorp LLC, College Station, TX).

Power analysis was conducted using the “ipdpower” command in STATA [38]. The ipdpower command was a simulation-based tool that was specially developed for power calculation for multi-level mixed effects models. We set the parameters at the sample size (n = 120) and clusters (n = 8) which were similar to the data structure in the current study. We specified the simulation (n = 1000) with random effects for the intercept, fixed treatment effects and fixed effects for the genotypes. Previous studies indicated the difference of BMI z-scores between the C allele carriers and noncarriers of the *CLOCK* SNP was around 0.4, and the difference between the AA allele carriers and TT allele carriers of the *FTO* SNP ranged from 0.2 to 1.1 [23–25, 39]. Therefore, we assumed a low effect size of 0.5 and a moderate effect size of 1 to conduct the power analysis. For the genetic association, our study had a power of 47% with the low effect and 96% with the moderate effect. For the interaction effect, our study had a power of 28% with the low effect and a power of 77% with the moderate effect. Based on the simulation results, our study would have low power if the SNPs had a low effect size on BMI z-scores and relatively adequate power if the SNP or the interactions had a moderate effect.

## Results

### Participants characteristics

At the baseline, with the exception of 2 boys and 1 girl, all youth were between 9 and 10 years of age. Boys’ mean age was 9.21 ± 0.53 years; Girls’ mean age was 9.08 ± 0.33 years.

The majority of our participants were white (81%, n = 98) and 56% (n = 68) were boys. Most of the children (83%, n = 100) spent no more than three hours daily on screen time. Obesity affected 6.6% (n = 8) of the children. The percentage of the children with the rs1801260 genotypes, AA, AG, and GG were 45% (n = 55), 44% (n = 53), and 11% (n = 13), respectively. The percentage of the children with the rs9939609 genotypes, TT, AT, and AA, were 39% (n = 47), 50% (n = 61), and 11% (n = 13), respectively. These characteristics were not significantly different between the intervention and the control groups at the baseline or the 12-month follow-up (Table 1). At the 12-month follow-up, 6.4% (n = 7) of the children were obese.

**Table 1:** Characteristics of youth participants.

	Intervention	Control	P*
**Baseline (n = 121)**	n = 60	n = 61	
Age (years, mean, sd)	9.12 (0.37)	9.18 (0.53)	0.45
Sex (male, %, n)	55% (33)	57% (35)	0.79
Race (white, %, n)	77% (46)	85% (52)	0.23
Screen time (≤3 hours/day, %, n)	85% (51)	80% (49)	0.5
BMI z-scores (mean, sd)	0.08 (0.94)	-0.08 (1.17)	0.4
Obesity^a^ (%, n)	5% (3)	8% (5)	0.48
Overweight^a^ (%, n)	7% (4)	8 (5)	0.75
rs1801260 (*G* allele, MAF^b^)	0.33	0.34	0.96
rs9939609 (*A* allele, MAF^b^)	0.38	0.34	0.62
**12 mo Follow-up (n = 109)**	n = 55	n = 54	
Age	10.67 (0.36)	10.59 (0.3)	0.19
Sex (male, %, n))	56% (31)	61% (33)	0.62
Race (white, %,n)	78% (43)	83% (45)	0.5
Screen time (≤3 hours/day, %, n)	87% (47)	76% (41)	0.13
BMI z-scores	0.06 (1.04)	-0.2 (1.26)	0.26
Obesity (%, n)	7.3% (4)	5.6% (3)	0.72
Overweight (%,n)	9% (5)	15% (8)	
rs1801260 (*G* allele, MAF^b^)	0.33	0.31	0.73
rs9939609 (*A* allele, MAF^b^)	0.36	0.36	0.97

*t test and chi-squared test were used to estimate the p values.

^a^Obesity and overweight were classified according to the CDC criteria.

^b^MAF is the minor allele frequency

### Gene-by-intervention interaction on BMI z-scores at the follow-up

The Fuel for Fun intervention did not significantly affect the children’s BMI z-scores at the follow up, when the baseline BMI z-scores and other covariates were controlled (*p* = 0.27). The *CLOCK* SNP, rs1801260, did not significantly modify the intervention effect on BMI z-scores at the follow-up (*p*_interaction = 0.38). Neither did rs1801260 modify the intervention effect on changes of BMI z-scores between the baseline and follow-up (*p*_interaction = 0.43). The *FTO* SNP, rs9939609, did not significantly modify the effect of the intervention on BMI z-scores at the follow-up (*p*_interaction = 0.16) or changes of BMI z-scores (*p*_interaction = 0.25).

### Gene-by-sex interaction on BMI z-scores at baseline and follow-up

**Fig 1:**
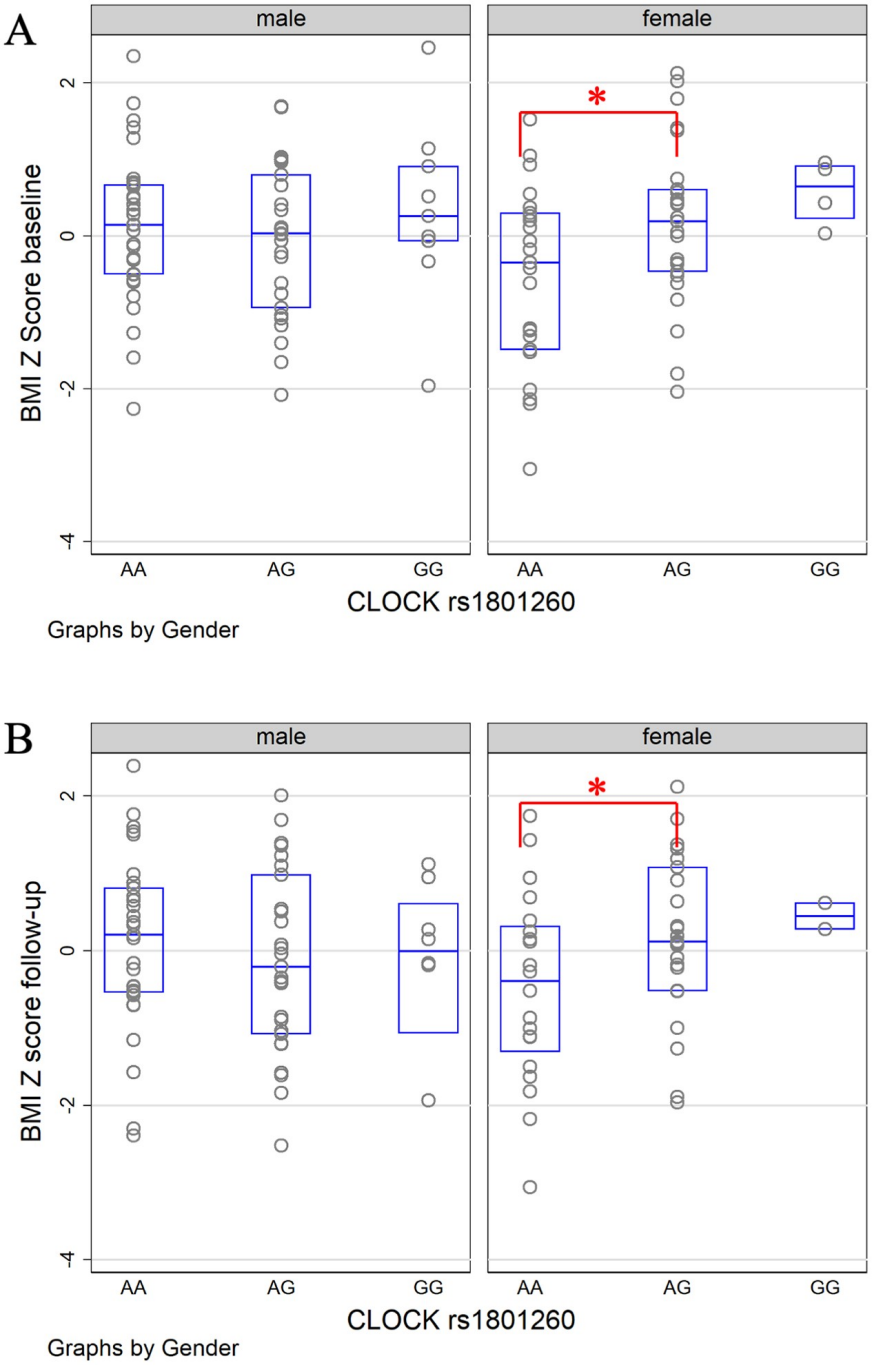
The distribution of standardized BMI z-scores by sex and rs1801260 genotypes at the baseline and 12-month follow-up. The figure displays the scatterplot and boxplot of the BMI z-scores. * indicates the significant difference between genotypes.

**Fig 2:**
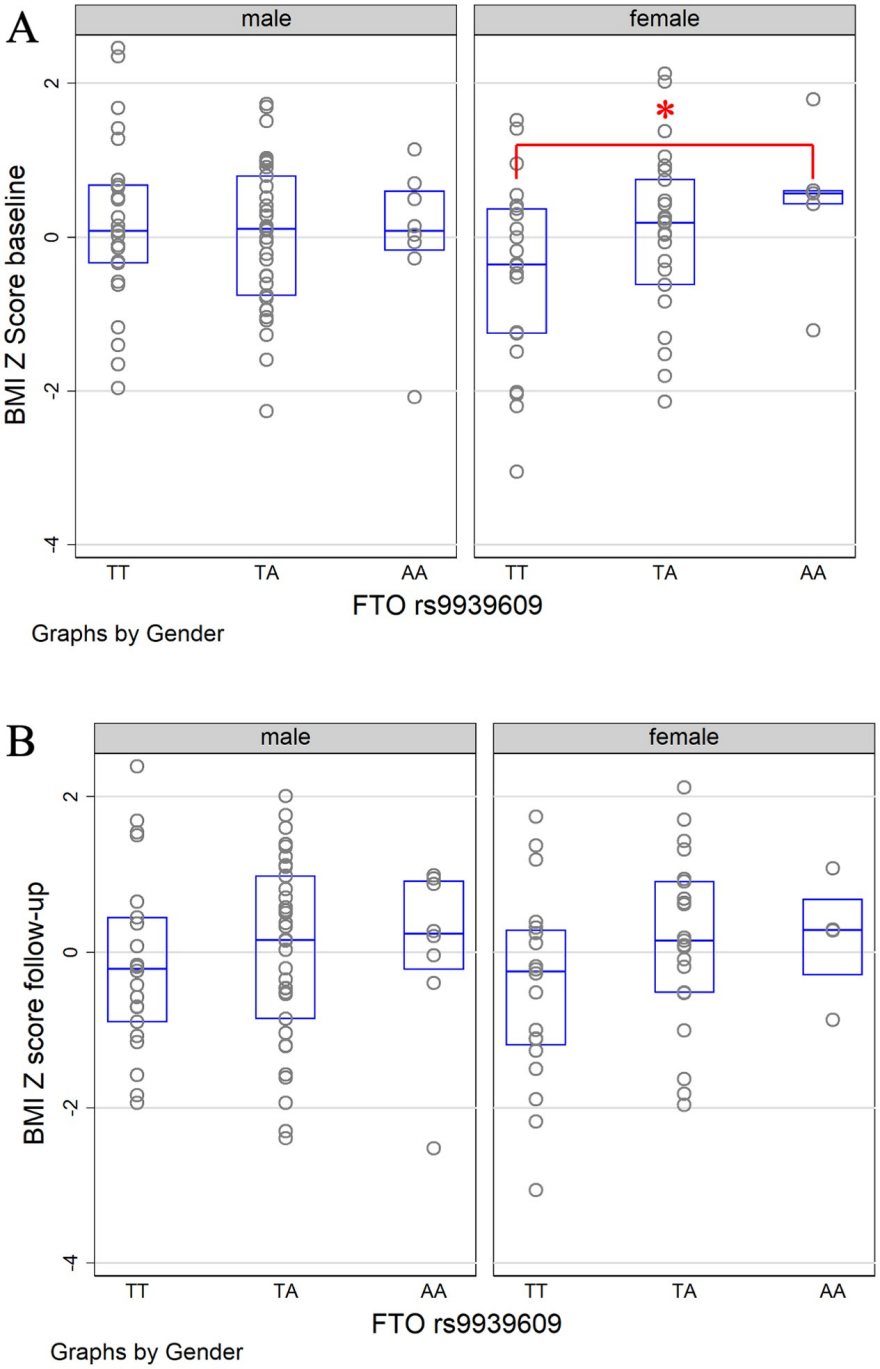
The distribution of standardized BMI z-scores by sex and rs9939609 genotypes at the baseline and 12-month follow-up. The figure displays the scatterplot and boxplot of the BMI z-scores. * indicates the significant difference between genotypes.

At the baseline, rs1801260 was not significantly associated with BMI z-scores (*p* = 0.31), neither was rs9939609 (*p* = 0.85). However, sex nominally significantly modified the association of rs1801260 with BMI z-scores (Table 2. *AG* vs *AA*: *p*_interaction = 0.03). Further stratified analyses between boys and girls showed that nominally significant genetic association with BMI z-scores was found in girls but not in boys (Fig 1A; in girls: *AG* vs *AA* coefficient = 0.6, *p* = 0.04, 95% CI: 0.02–1.18; *GG* vs *AA* coefficient = 1.33, *p* = 0.02, 95% CI: 0.17–2.49). Sex also nominally significantly modified the association between BMI z-scores and rs9939609 when the additive effect of the *A* allele was considered (*p* = 0.03). This additive effect of rs9939609 was only nominally significant in girls (*p* = 0.049, Fig 2A).

**Table 2:** The modification effect of sex on the association between the *CLOCK* SNP, rs1801260, and BMI z-scores.

		BMI z-scores			
		Coefficient	95% confidence interval		P
**Baseline**					
rs1801260	*GG*	0.151	-0.624	0.926	0.703
	*AG*	-0.191	-0.730	0.348	0.488
	*AA*	Reference			
Sex _girl		-0.645	-1.208	-0.082	0.025
Sex × rs1801260	*GG*	0.787	-0.579	2.152	0.259
	***AG***	**0.879**	**0.091**	**1.667**	**0.029**
	*AA*	Reference			
**Follow-up**					
rs1801260	*GG*	-0.323	-1.220	0.573	0.479
	*AG*	-0.305	-0.923	0.313	0.333
	*AA*	Reference			
Sex _girl		-0.585	-1.242	0.072	0.081
Sex × rs1801260	*GG*	1.306	-0.615	3.226	0.183
	***AG***	**1.069**	**0.137**	**2.001**	**0.024**
	*AA*	Reference			

Linear mixed-effects models adjusted for age, sex, race, screen time, and family and school clusters. At the follow-up, assigned nutrition education group was also controlled in the model.

At the follow-up, intervention groups were also adjusted in the analysis. rs1801260 was again not significantly associated with BMI z-scores (*p* = 0.58), neither was rs9939609 (*p* = 0.77). Sex also nominally significantly modified the association of rs1801260 with BMI z-scores at the follow-up (Table 2. *AG* vs *AA*: *p*_interaction = 0.02). Similarly, nominally significant genetic association with BMI z-scores was found in girls but not in boys (Fig 1B; in girls: AG vs AA coefficient = 0.65, *p* = 0.02, 95% CI: 0.12–1.17; GG vs AA coefficient = 1.04, *p* = 0.2, 95% CI: -0.55–2.62). However, this interaction between sex and the SNP became insignificant (*AG* vs *AA*: *p*_interaction = 0.57), when the baseline BMI z-scores were also controlled in the model. Neither did sex modify the association between rs1801260 and changes of BMI z-scores between the baseline and follow-up (*p*_interaction = 0.39). The interaction between sex and rs9939609 did not reach significance for the follow-up BMI z-scores (*p*_interaction = 0.24, Fig 2B) even when the baseline BMI z-scores were not controlled or for the changes of BMI z-scores (*p*_interaction = 0.17).

Function analysis using HaploReg indicated that rs1801260 resides in the cis-regulatory elements, including enhancer and DNase I-hypersensitive site. The SNP, rs9939609, and particularly SNPs in linkage disequilibrium also reside in the cis-regulatory elements. Analysis using the GTEx Portal indicated that rs1801260 potentially affects the expression level of the *CLOCK* gene. But rs9939609 may not affect the expression level of the *FTO* gene.

## Discussion

This study explored the potential association of a *CLOCK* SNP and a *FTO* SNP with BMI z-scores and their modification effect on the culinary nutrition and physical activity intervention in a group of school-age children. Sex was found to nominally significantly modify the association between rs1801260 and BMI z-scores, although this SNP did not modify the effect of our culinary nutrition and physical activity intervention on BMI z-scores. In girls, the carriers of the risk allele (G) was associated with higher BMI z-scores at the baseline and the 12-month follow-up. These results indicate that the *CLOCK* gene may play different roles in the development of obesity in school-age boys and girls.

The SNP, rs1801260, is located at the 3’-untranslated gene region of the *CLOCK* gene. Function analysis indicates that this SNP resides in the cis-regulatory elements and consequently is found to regulate the transcription of the *CLOCK* gene. Previous studies have linked this SNP with sleep, feeding behaviors, glucose intolerance and autonomic nervous function [16, 17, 20, 40].

Thus far, few studies have assessed the association of the *CLOCK* gene with obesity in the pediatric population. Giovaninni et al. (n = 340) [23] and Valladares et al. (n = 256) [24] both assessed the relationship of rs1801260 with BMI in school-age children. Although the association did not reach statistical significance, children who carried the minor allele had a higher rate of overweight and/or BMI z-scores in their studies. Similarly, our study did not find a significant main effect of the SNP on BMI z-scores. However, school-age girls with the minor allele had higher BMI z-scores compared to the girls carrying homozygous major allele. The modification effect of sex was also found in a study with elderly adults [12]. In this group of menopausal women, rs1801260 was possibly associated with overweight/obesity. But contrary to our findings, the minor allele was linked to a lower risk of obesity. The main difference between the two studies may be a result of the differences in developmental stages and sex hormone levels such as estrogen. The majority of our participants were between 9 and 10 years old at the baseline assessment, an age associated with gradual increases of the estrogen (e.g., estradiol and estrone) level [41]. In postmenopausal women, estradiol and estrone concentrations are significantly reduced [42]. The estradiol level has already been recognized to regulate glucose and lipid metabolism and food intake [43, 44]. Therefore, it is possible that the difference in estrogen level between peripubertal girls and menopausal women may moderate the association between the *CLOCK* SNP and BMI, as the *CLOCK* gene also plays a role in lipid metabolism and dietary intake [3–5].

With regard to the minor allele (G) of rs1801260, several studies of this SNP in the general adult population and school-age children identified a similar effect as did the current study, which suggest the minor allele of rs1801260 is linked to higher BMI [10, 14, 15, 23, 24]. The minor allele of rs1801260 was related to the expression level of the *CLOCK* gene and consequently might affect the *CLOCK*-related obesogenic mechanism. Studies have shown that the minor allele of this SNP was associated with lower adiponectin concentration, shorter sleep duration, higher plasma ghrelin concentrations, higher saturated fat intake, and lower response to satiety [10, 16, 17, 24]. Therefore, further research is warranted to clarify the effect of the *CLOCK* SNP, rs1801260, and examine the relationship between sex and the *CLOCK* gene particularly in the pediatric population.

The SNP, rs9939609, is within the first intron of the *FTO* gene. Although it may not affect the expression level of the *FTO* gene, it is potentially related to the *IRX3* gene and likely plays a role in regulating metabolic rate in the adipose tissue [31]. The association between this SNP and BMI z-scores did not reach statistical significance. Interestingly, previous studies have shown that age strengthens the association between this SNP and BMI in pediatric population [45, 46]. In children younger than 11 or 10 years old, the association between the *FTO* SNP and BMI was not significant. This association predominated until children reached 11–12 years of age. Thus, the relatively younger age of the children in our study may explain the lack of association of rs9939609 with BMI according to the tendency of previous studies. In addition, sex might modify this association, although the results were not consistent in our study; in girls rs9939609 was only associated with the baseline BMI z-scores. The sex-dependent association of rs993969, especially in girls has been reported previously [39, 46]. Further research is necessary to confirm the age- and sex-dependent association of the *FTO* variants with BMI in pediatric samples.

Our study first identified that sex potentially modified the association of the *CLOCK* SNP, rs1801260, with childhood obesity. But several limitations need to be taken into consideration. The major limitation is lack of measures of sleep quality and dietary intake. With these assessments, studies could further evaluate potential obesogenic mechanisms involved in the candidate genes. Another limitation is that the pubertal status and sex hormone (such as estrogen) were not assessed. This study found that sex potentially modified the association between the *CLOCK* SNP and BMI z-scores in school-age children. Further assessment of puberty and sex hormones may explain this modification effect of sex. Additionally, the sample size of this study had limited power to identify genes with low effect size, so the non-significant results need to be interpreted cautiously. Future studies with larger sample size potentially have the ability to specify the main effect of these SNPs in children. Also, only one *CLOCK* SNP was analyzed in our study. Additional *CLOCK* SNPs and other circadian genes need to be investigated in the pediatric population to delineate the association of circadian system with the development of childhood obesity.

The current study suggests that sex is a potential modifier for the relationship between the *CLOCK* gene and BMI z-scores in school-age children. Further investigation is warranted to outline the relationship among pubertal development, sex hormones and the *CLOCK* polymorphisms to reveal potential obesogenic mechanisms involved in the disruption of circadian rhythms.

## Supporting information

S1 Data (XLS)
